# Supplementary material for: Coordination games in cancer
Source: PLoS One. 2022 Jan 21;17(1):e0261578. doi: 10.1371/journal.pone.0261578 (PMC8782377; doi:10.1371/journal.pone.0261578)
Supplement: S1 File — (PDF) [file pone.0261578.s001.pdf]

Supplementary information for ‘Coordination games in cancer’[1]

This supplementary material contains three technical discussion points: the mathematical conditions for coordination games to emerge, how ‘coordination-like’ games could arise from non-coordination games through assortative matching within the interactions of cancer cells, and the biological interpretation of the convexity parameter α.

S1. Coordination games conditions

Evolutionary games with two strategies need to meet two sufficient and necessary conditions to produce a coordination game:

1. Each strategy proliferates at a higher rate in its own environment than in the others’ environment.

2. Neither strategy dominates the other.

Both these conditions are mild in terms of their biological implications. The former requires that the strategies be ‘selfish’, a concept that is very well documented in evolutionary biology. The latter requires that neither strategy be more fit than the other in both environments. If one strategy were to dominate the other, then the dominant strategy invariably would outcompete the dominated one.

Using the the payoff parameters r_1, r_2, d_1, d_2 as in Table 1, and assuming $r_1 \geq r_2$ without loss of generality, (1) can be expressed as $r_1 \geq d_1$ and $r_2 \geq d_2$, while (2) amounts to assuming $r_2 \geq d_1$, as $r_1 \geq d_2$ already follows from (1).

Under these assumptions the game will have three Nash equilibria: purely type 1, purely type 2, and a mixed equilibrium where the frequency of type 1 equals g^*, which can be obtained by setting type 1’s payoff equal to type 2’s as follows:

$$g^* r_1 + (1 - g^*) d_1 = (1 - g^*) d_2 + g^* r_2,$$

yielding $g^* = \frac{r_2 - d_1}{r_1 + r_2 - d_1 - d_2}$, which is between 0 and 1 due to assumptions (1) and (2).

Due to (1) and (2), we have $r_2 \geq d_1$, meaning that strategy 1 cannot invade the purely type 2 equilibrium, while the assumption $r_1 \geq r_2$, together with (1) implies that $r_1 \geq d_2$, hence strategy 2 also cannot invade the purely type 1 equilibrium, meaning that both pure equilibria constitute evolutionary stable strategies. On the other hand, the mixed equilibrium is unstable due to $g^* r_1 + (1 - g^*) d_2 \leq r_1$ and $(1 - g^*) r_2 + g^* d_1 \leq r_2$, hence both strategies are able to invade it. Under typical imitation dynamics, the mixed equilibrium separates the basins of attraction of the two pure equilibria, populations with frequencies of type 1 larger than g^* converge to the pure type 1 equilibrium, while those lower than g^* converge to the pure type 2 equilibrium.

[1]by Péter Bayer, Robert A. Gatenby, Patricia H. McDonald, Derek R. Duckett, Kateřina Staňková, and Joel S. Brown. Preprint available on BioRxiv at https://www.biorxiv.org/content/10.1101/2021.06.22.449436v1.

		Predominant strategy	
		Type 1	Type 2
Focal player	Type 1	r_1	d_1
	Type 2	d_2	r_2

Table 1: An evolutionary game with two strategies is a coordination game if conditions (1) and (2) are satisfied, that is $r_1 \geq d_1$, $r_2 \geq d_2$, and $r_2 \geq d_1$, with $r_1 \geq r_2$ assumed without loss of generality. Alternatively, assortative matching can also produce 'coordination-like' games.

We highlight that our condition 1, particularly $r_2 \geq d_2$, is not standard in the characterizations of coordination games, despite all canonical coordination games (pure coordination, choosing sides, stag hunt) satisfying it. The minimal characterization of non-cooperative coordination games only requires that all symmetric strategy profiles be Nash equilibria. As the direction of this inequality has no bearing on the equilibrium structure, usually, no assumption is made on it. For our story it is sensible to restrict our attention to the case where any deviation from a coordinated strategy profile is harmful for all players. Nevertheless, all qualitative results and observations of our paper go through without relying on this assumption.

S2. Coordination games through assortative matching

A second avenue in which 'coordination-like' games may arise is through assortative matching in a game that is itself not necessarily a coordination game. In the current context, assortative matching means that a player (cancer cell) with a given strategy is more likely to interact with players of the same strategy.

For example, in a Prisoner's Dilemma game with assortative matching, if cooperators are sufficiently more likely to interact with other cooperators, then cooperation is an evolutionary stable strategy. If, however, the assortative matching is not too strong, then desertion is also evolutionary stable.

To formalize this, take $d_2 > r_1 > r_2 > d_1$, which characterizes a Prisoner's Dilemma game. In this case, neither (1) nor (2) are satisfied. Let σ denote the the rate of assortative matching, i.e. the probability of interaction with a player (cell) of the same type. With probability $1 - \sigma$, the player interacts with an opponent chosen from a well-mixed sample of the population. Then, $\sigma > (d_2 - r_1)/(d_2 - r_2)$ will mean that Strategy 1 (Cooperation) is evolutionarily stable as

$$r_1 > \sigma r_2 + (1 - \sigma) d_2,$$

meaning that Strategy 2 (Defection) cannot invade Strategy 1.

Similarly, if $\sigma < (r_2 - d_1)/(r_1 - d_1)$, then Type 2 (Defection) is evolutionarily stable as

$$r_2 > \sigma r_1 + (1 - \sigma) d_1,$$

thus, Strategy 1 also cannot invade Strategy 2, producing the two pure evolutionary stable strategies of coordination games.

A σ that is able to satisfy both conditions exists if and only if $r_1 + r_2 > d_1 + d_2$. Therefore, 'coordination-like' games arise from the more widely studied Prisoner's Dilemma game even if conditions (1)-(2) are not met but the sum of main-diagonal elements of the payoff matrix shown in Table 1 is larger than the sum of off-diagonal elements. The frequency of type 1 cells in the unstable mixed equilibrium that separates the basins of attraction is again denoted by g^*. It is calculated, as before, by setting the payoffs of the two types equal as follows:

$$\sigma r_1 + (1-\sigma)\left(g^* r_1 + (1-g^*)d_1\right) = \sigma r_2 + (1-\sigma)\left((1-g^*)r_2 + g^* d_2\right),$$

leading to

$$g^* = \frac{\frac{\sigma}{1-\sigma}(r_2 - r_1) + r_2 - d_1}{r_1 + r_2 - d_1 - d_2}.$$

The main take-away from this exercise is to showcase that, although as we argue, the conditions under which coordination games can arise are mild, even when those conditions are not met, standard models of assortative matching lead to the emergence of 'coordination-like' games with identical transient dynamics.

S3. Convexity as a result of cell interactions in groups

Our numerical examples of cancer growth and treatment all the underlying games were convex ($\alpha = 2$). The extreme specifications are linear games ($\alpha = 1$) in which discoordination is punished in proportion to the frequency of discoordinating players, and the extremely stringent punishments where any amount of discoordination is penalized the same way (0 payoffs if not all players agree, amounting to $\alpha \to \infty$). The former calibration would reflect the spirit of classical evolutionary games which assumes pairwise interactions between players in a well mixed population, the latter one reflects a pure coordination game played by the entirety of the population. Our calibration strikes a balance between these two extremes but our qualitative results are not sensitive to variations in α.

The biological interpretation of the $\alpha = 1$ calibration is straightforward and largely conforms to the standard one in evolutionary game theory, in which individuals (in our case, cancer cells) are well-mixed and perform pairwise interactions. Thus, if the composition of the tumor is g, the probability of encountering a type 1 cell is g, and the probability of encountering a type 2 cell is $1-g$. Thus, for type 1 and type 2, respectively, the expected values of interacting with a randomly chosen cell of the population are given as

$$\pi_1(g) = g r_1 + (1-g)d_1,$$

$$\pi_2(g) = (1-g)r_2 + g d_2,$$

producing the growth terms of our model for $\alpha = 1$.

For $\alpha > 1$, the biological interpretation of the growth rates in our model is more complex but it is also rooted in the concept of cellular interactions, as long as α is integer-valued. Instead of cells

meeting pairwise at random, each cell interacts with n others, with $n > \alpha$, sampled at random from the well-mixed tumor population. Thus, cancer cells interact in groups of $n+1$, and a cell's value of these interactions is dependent on the cell's own type and the composition of its group. The value of a type 1 (type 2) cell interacting with a group that has k other type 1 cells is denoted by a_k (b_k). Then, for types 1 and 2, respectively, the expected values gained from an interaction of a group of size n sampled from a large, well-mixed population where the fraction of type 1 cells is x are given as

$$\pi_1(g) = \sum_{k=0}^{n} \binom{n}{k} g^k (1-g)^{n-k} a_k,$$

$$\pi_2(g) = \sum_{k=0}^{n} \binom{n}{k} g^k (1-g)^{n-k} b_k.$$

We note that $\pi_1(g)$ and $\pi_2(g)$ are polynomials in Bernstein form of degree n with Bernstein coefficients $(a_k)_{k=0}^n$ and $(b_k)_{k=0}^n$. Let the payoff series a_k be given as the sum of two series, $a_k = a_k(r_1) + a_k(d_1)$ with:

$$a_k(r_1) = \begin{cases} 0 & \text{if } k < \alpha, \\ r_1 \frac{\binom{k}{\alpha}}{\binom{n}{\alpha}} & \text{if } k \geq \alpha, \end{cases} \tag{1}$$

and

$$a_k(d_1) = \begin{cases} d_1 \frac{\binom{n-k}{\alpha}}{\binom{n}{\alpha}} & \text{if } k \leq n-\alpha, \\ 0 & \text{if } k > n-\alpha. \end{cases} \tag{2}$$

With this calibration, the Bernstein polynomial $\pi_1(g)$ reduces to

$$\pi_1(g) = g^\alpha r_1 + (1-g)^\alpha d_1,$$

as the Bernstein polynomial with coefficients $a_k(r_1)$ equals $g^\alpha r_1$ while the one with coefficients $a_k(d_1)$ equals $(1-g)^\alpha d_1$. Thus, the value of interaction matches the type 1's growth rate in our paper's model.

From very similar considerations, letting $b_k = b_k(r_2) + b_k(d_2)$ with

$$b_k(r_2) = \begin{cases} r_2 \frac{\binom{n-k}{\alpha}}{\binom{n}{\alpha}} & \text{if } k \leq n-\alpha, \\ 0 & \text{if } k > n-\alpha, \end{cases} \tag{3}$$

and

$$b_k(d_2) = \begin{cases} 0 & \text{if } k < \alpha, \\ r_1 \frac{\binom{k}{\alpha}}{\binom{n}{\alpha}} & \text{if } k \geq \alpha, \end{cases}, \tag{4}$$

will give

$$\pi_2(g) = (1-g)^\alpha r_2 + g^\alpha d_2.$$

The interpretation of the payoff series a_k is the following: The component $a_k(r_1)$ contains the gains of coordination of a type 1 cell. A type 1 cell receives positive value from the group interaction as long as the number of type 1 cells within the sample is at least α. The value increases with the number of

type 1 cells, reaching a maximum of r_1 if every cell in the interaction group is of type 1. The component $a_k(d_1)$ contains the gains of discoordination of a type 1 cell. The cell receives positive value from the group interaction as long as the number of type 2 cells is at most α. The value increases with the number of type 2 cells, reaching a maximum of d_1 if every cell in the interaction group is of type 2. The cell's total value of the interaction is the sum of the coordination gains and the discoordination gain. The interpretation of the series b_k and its components is identical.

With $d_1 = d_2 = 0$, the game is a pure coordination game and positive gains are only attainable for cells if their group has at least α cells of the same type. Then, the cells' growth rates are monotonic functions of the number of type 1 cells in their interaction group, increasing for a type 1 cell and decreasing for a type 2. If d_1 and d_2 are positive, then both growth rates are U-shaped in the number of type 1 cells in their sample: either extreme is better than a heterogeneous mix. Figure 1 shows the payoff series a_k with $n = 100$ and $\alpha = 1, 2, 5, 10, 25, 50, 95$ for these two games.

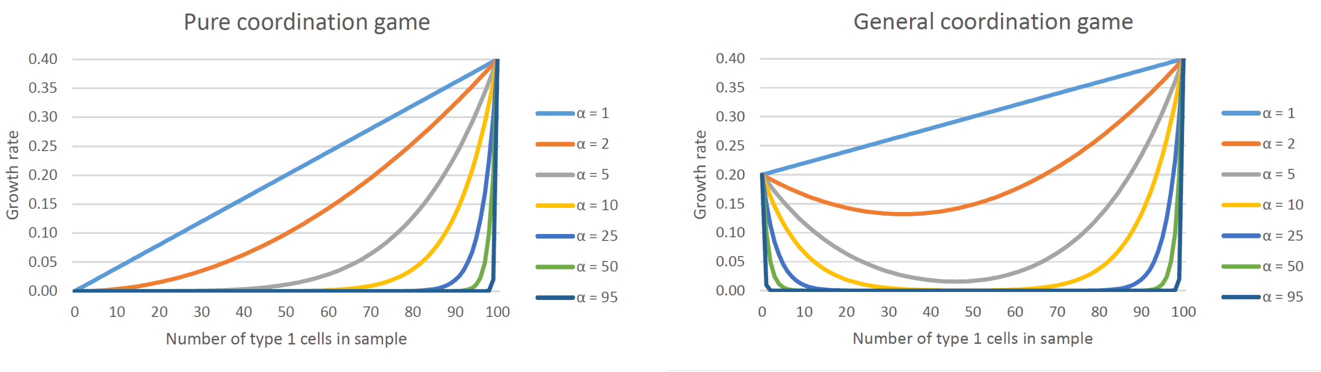

Figure 1: The payoff series a_k under various values of α in the pure coordination game (left) and a general coordination game with positive discoordination payoff (right). Parameters: $r_1 = 0.4$, $d_1 = 0$ (left), $d_1 = 0.2$ (right).

S4. Adaptive therapies

We consider four adaptive therapies in convex coordination games; one high-amplitude, and three low-amplitude therapies at a small population, medium population, and large population, respectively. In our example, each plan of adaptive therapy is able to keep control of the tumor and each offers a different set of advantages an disadvantages. Setting the lower bound at a small population produces a therapy with longer treatment cycles. Practically, this is beneficial but a low lower bound also means the tumor composition approaches the resistant type's basin of attraction, risking losing control over the tumor. Setting a high upper bound gives the tumor more time to recover its composition which lowers the risk of losing control but exposes the patient to periods of high tumor burden. For a given amplitude of on-off cycling, deciding between a planned therapy aimed at maintaining small, medium, or a large population results in a tradeoff between the frequency of cycles, the risk of losing control, and periods of high tumor burden. In Table 2 we report these tradeoffs explicitly for the four adaptive

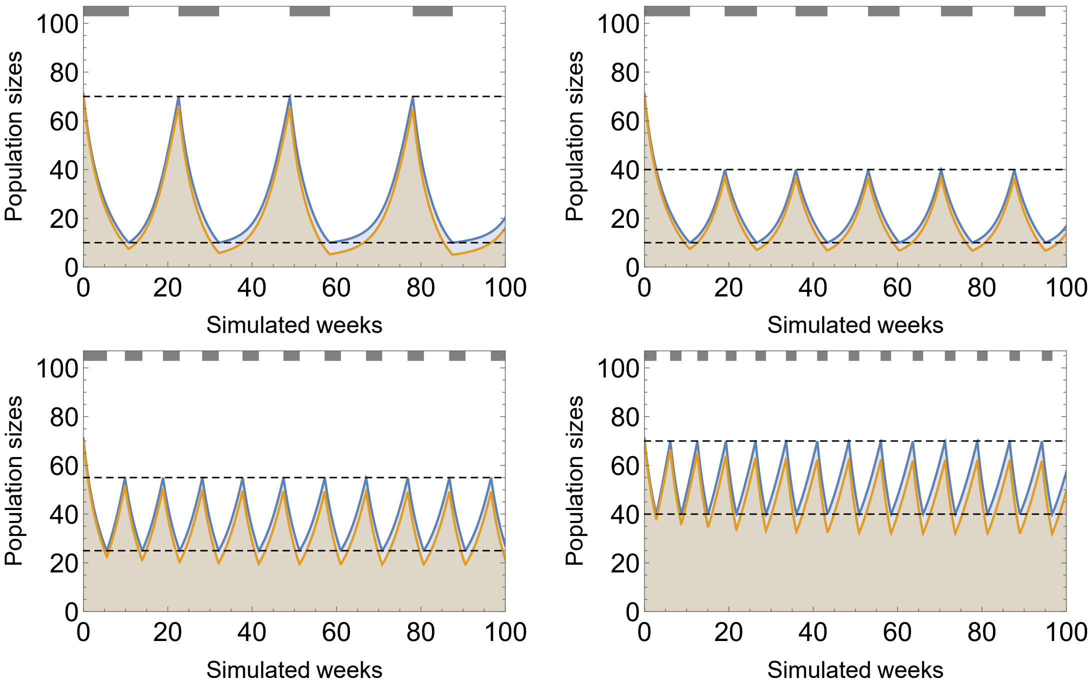

Figure 2: Four adaptive therapy strategies: *high amplitude* (top left), *low amplitude at small population* (top right left), *low amplitude at medium population* (bottom left), and *low amplitude at large population*. The tumor is kept under control indefinitely in each one. Parameters: $r_1 = 0.4$, $r_2 = 0.2$, $d_1 = d_2 = 0$, $K = 150$, $c_1 = c_2 = 0.05$, $m_1 = m_2 = 0.01$, $\alpha = 2$, $\gamma_1 = 0.4$, $\gamma_2 = 0$.

treatment strategies considered in Figure 2.

Treatment strategy $(\underline{x}, \overline{x})$	Length of cycles (Sim. weeks)	Maximum tumor burden	Minimum composition (%)
High-amplitude (10, 70)	31	70	49
Low-amplitude, small pop. (10, 40)	17	40	66
Low-amplitude, medium pop. (25, 55)	10	55	77
Low-amplitude, large pop. (40, 70)	8	70	80

Table 2: The characteristics of the four adaptive treatment strategies considered in Figure 2. High-amplitude adaptive therapy produces long cycles at the cost of approaching the resistant type's basin of attraction and periods of high tumor burden. Low-amplitude therapies trade off the length of treatment cycles, which are the longest at small population levels, against the risk of losing control, which is lowest at large population level.
