# Supplementary material for: Coordination games in cancer
Source: PLoS One. 2022 Jan 21;17(1):e0261578. doi: 10.1371/journal.pone.0261578 (PMC8782377; doi:10.1371/journal.pone.0261578)
Supplement: Attachment — Submitted filename: Response letter.docx [file pone.0261578.s002.docx]

Dear Editor and Reviewers,

We would like to express our gratitude for your efforts in reading and commenting on our paper. Your feedback has been highly valuable and contributed greatly to improving our paper. Below is a list of responses to the specific comments.

Reviewer 1:

**One point, which I must suggest the authors when revising to a revised MS, is that Introduction part should contain some important recent findings of evolutionary game theory. That is; the universal concept of dilemma strength in 2 by 2 game including coordination game.**

**As the authors saying the current MS, what some previous studies presuming to adopt the game framework into tumor cell growth is Prisoner’s dilemma (PD) or Chicken game (other favor to call Snowdrift game). Needless to say, a coordination game belongs to Stag Hunt (SH) game. In general for a symmetric 2 by 2game, there are two dilemmas; Chicken-type dilemma (of which extent is quantified by Dg’ := (T – R)/(R – P)) and Stag Hunt-type dilemma (of which extent is quantified by Dg’ := (P – S)/(R – P)), PD is simultaneously suffered from both dilemmas (Dg’ >0 & Dr’>0). In contrast, SH game is feature only with Stag Hunt-type dilemma; Dg’<0 & Dr’>0.**

**I strongly suggest the authors to review the concept of dilemma strength in view of ‘coordination game’ in Introduction part by citing some relevant literature; (i) Dilemma strength as a framework for advancing evolutionary game theory: Reply to comments on “Universal scaling for the dilemma strength in evolutionary games”, Physics of Life Reviews 14, 56-58, 2015, (ii) Scaling the phase- planes of social dilemma strengths shows game-class changes in the five rules governing the evolution of cooperation, Royal Society Open Science, 181085, 2018, (iii) Social efficiency deficit deciphers social dilemmas, Scientific Reports 10, 16092, 2020.**

We thank you for this very interesting suggestion. This literature allowed us to frame our coordination games better. We’ve inserted the following text on page 2, starting on line 63 (please note that minor differences in line numbers may occur between the unmarked and the marked manuscript, here we provide line references for the marked manuscript):

*“A recent strand of literature classifies 2 x 2 evolutionary games of cooperation and defection based on dilemma strength (Wang et al., 2015; Ito and Tanimoto, 2018; Arefin et al., 2020). The ‘Gamble-Intending Dilemma’ (GID) measures the gains a player would realize by defecting rather than cooperating if the opponent cooperates (e.g., in a prisoner’s dilemma game, being released from prison). The ‘Risk-Averting Dilemma’ (RAD) measures the losses a player can avoid by defecting rather than cooperating if the opponent defects (e.g., an extended prison sentence). The two measures can be positive or negative, giving rise to four canonical game classes: If both are positive, defection dominates as in the prisoner’s dilemma, if GID is positive but RAD is negative, the game is an anti-coordination game and the dilemma is that of a chicken game (a.k.a. hawk-dove game or snowdrift game), whereas if GID is negative and RAD is positive, it is a coordination game and the dilemma resembles that of a stag hunt game. If both are negative, cooperation dominates, characterizing ‘Harmony’, that is, the absence of a social dilemma.*

*Coordination games capture situations without an explicit definition of cooperation or defection. In the purest case, neither strategy is socially optimal, the only thing that matters is whether the players agree. In the more general case, the strategies are asymmetric in terms of efficiency (the payoffs of players in case of coordination) and risk (the payoffs of discoordinating players).”*

As a terminological note: Usually in non-cooperative game theory, coordination games are considered to be a superset of Stag Hunt games, not the other way around. This is due to the fact that the common definition of coordination games only requires that all symmetric pure strategy profile be Nash equilibria, which is a property that all Stag Hunt games satisfy.

In our manuscript, we require something stronger, we require that any deviation from a symmetric strategy profile be harmful for all individuals. In the notation introduced by the reviewer, this amounts to having $R>S$. This does not change the game’s equilibrium structure, but highlights the incompatibility between the phenotypes. As this is an important distinction to common definitions of coordination games, we highlight this in our Supplementary Information 1, ‘Coordination games conditions’. We thank the reviewer for bringing our attention to this terminological detail. As most of the canonical coordination games have this feature, we feel our extra assumption does not require a new terminology and we can stick with ‘coordination games’.

*“We highlight that our condition 1, particularly* $r_{2}\geq d_{2}$*, is not standard in the characterizations of coordination games, despite all canonical coordination games (pure coordination, choosing sides, stag hunt) satisfying it. The most minimal characterization of non-cooperative coordination games only requires that all symmetric strategy profiles be Nash equilibria. As the direction of this inequality has no bearing on the equilibrium structure, usually, no assumption is made on it. For our story it is sensible to restrict our attention to the case where any deviation from a coordinated strategy profile is harmful for all players. Nevertheless, all qualitative results and observations of our paper go through without relying on this assumption.”*

Reviewer 2:

Comment 1:

**I cannot understand the validity of the model.**

**In both the lung and breast cancer explanations, it is unclear whether game-like dynamics really exist in the background. In other words, I don't think these explanations provide examples of what cancer dynamics this model can be applied to. These sentences are argumentative and don't seem to motivate the development of the model.**

We agree, in part, with the reviewer’s concern. We offer these two cancers and the role of driver mutations as possible examples of coordination games occurring in cancer. That there are reasons to believe that such game occur in cancer is what motivates the model. So that we acknowledge clearly the reviewer’s concern and our enthusiasm for still pursuing such a model we have added the following paragraph to the end of the section 2.1 Possible coordination games in cancer, page 5, starting on 181:

*“At this stage, we do not know for certain whether these two examples represent examples of coordination games. They do satisfy necessary conditions in that within a patient there is a predominant type and this predominant type differs between patients that have cancers derived from exactly the same tissue of origin. To be a coordination game, there must be frequency-dependent interactions where the fitness to a cancer cell type depends upon its neighbors’ cell types in a manner where being the rare subtype is highly disadvantageous. Once a lung cancer is dominated by EGFR-mut cells has a very different immune (low immune infiltration) and extra-cellular matrix than a KRAS-mut lung cancer with high immune infiltration and less structured extra-cellular matrix. An EGFR-mut cancer cell with its concomitant co-adaptations may find itself greatly disadvantaged within the tumor microenvironment of a KRAS-mut cancer and vice-versa. The same frequency-dependence seems to apply to breast cancer patients where just one or the other β-arrestin is upregulated but not both.”*

**The author also wrote as follows:**

***“The alternative strategies that the population could have coordinated upon are no longer visible. If coordination games exist in cancer, by the time the disease is detected, most cancer patients would likely present with a cancer that has already evolved to a common phenotype.”***

**Does above sentences mean that we don't know if there is a coordination game going on in cancer? In addition, it seems that at the time a cancer is discovered in the patient, a single type of cancer is already dominant and “Treatment with two types of competitive systems” as the authors assume is not possible. If so, what is the purpose of this model?**

The reviewer is correct. There are some excellent candidates for coordination games in cancer, especially when the subtypes of a cancer (such as lung cancer and others) can from different driver mutations and the subsequent co-adaptations. EGFR mutant and RAS mutant lung cancers exhibit different sets of highly mutated and different sets of highly conserved genes that result in quite different subtype-specific tumor microenvironments. But, in the absence of theory and a reason to look for them, they have not been tested nor proven to exist. (see the above paragraph that we have added)

We see the reviewer’s point regarding the pointlessness of proposing a therapy based on alternative strategies in a coordination game if just one strategy is all that will be present. This, of course, would be true if say strategy A is 100% of the cancer population. Such rarely, if ever, occurs in cancer cell populations that number in the millions in small tumors and 10’s of billions in large ones. Recurrent mutations, recurrent heritable epigenetic changes, and other mechanisms for generating heritable phenotypic variation ensure that there are a large number of low frequency and often less fit cancer cell types within a tumor. One reason for the evolution of resistance is the occurrence of resistant cancer cells at low frequency, even when there is a cost of resistance. We feel there is good reason to believe that alternative strategies of just about any cancer evolutionary game, including coordination games, will exist at low frequency or recur as heritable variation.

To raise both the caveat and the potential for such variation we have added the following to the initial paragraph of Section 3, page 7, line 233.

*“In each case, as expected, one of the strategies has become dominant in the patient’s tumor. Furthermore, we assume that the alternative strategy occurs at some very low frequency within the tumor as a result of recurrent mutations, epigenetic changes or other mechanisms for generating heritable phenotypic variation (Hu et al., 2017). This key assumption has empirical merit given how mutator phenotypes are a hallmark of cancer, and the occurrence of large amounts of genetic variability has been shown to exist in virtually all tumors (Greaves and Maley, 2012, Liguoro et al., 2020, Wang et al., 2020). Furthermore, selection on cancer cells lines in vitro results in rapid evolution of novel phenotypes (Han et al., 2016, Andrei et al., 2020).”*

Comment 2.

**In introduction section,**

***“As a result, the various strategies of these games are more likely to manifest between rather than within patients.”***

**I cannot catch up the meaning of this sentence.**

We have rewritten and expanded the sentence on page 3, line 98 to read:

*“Once clinically detectable, a single strategy from among those possible for the coordination game may have already become fixed within a patient. Thus, the different possible strategies of a coordination game are likely to manifest between rather than within patients. Furthermore, early driver mutations that can vary between patients with the same type of cancer offer a promising starting point for seeking these different strategies. The driver mutation itself along with associated coadapted genes and gene expression may be the principle strategy of a coordination game in cancer.”*

Comment 3.

**I am skeptical that the current title adequately describes this study, because the current model looks like a traditional LV model of 2-species competition system. Where is the game in that? The elements of payoff matrix are just used as part of the parameters of the LV model.**

Lotka-Volterra equations describe one of the most classical game dynamics, because they describe frequency-dependent selection. The first game dynamics describing evolution of behavioral strategies were introduced by Taylor and Jonker (1978) and among others were studied in detail by Hofbauer et al. (1979), Schuster et al. (1980, 1981), and Zeeman (1980, 1981). The replicator dynamics serve to model not only game dynamics, but also other biological processes occurring in population genetics and elsewhere. As Hofbauer (1981) showed, the replicator equation for n strategies corresponds - up to a change in velocity - to the generalized Lotka-Volterra equation for n-1 populations. Therefore, both replicator and Lotka-Volterra dynamics are game-theoretic. See also Wölfl et al (2021).

Comment 4.

**Furthermore, I felt that the author ignored the vast amount of knowledge and previous studies on the LV competition equation. As far as I know that LV competition model represent “competitive exclusion principle”, where we can see the elimination of species and the survival of only one species without the assumption of coordination game concept. Therefore, from the perspective of ecology, which has been trying to understand coexistence mechanisms, this model seems to be a derivative of many previous multispecies coexistence models. So, I have not yet to discover the uniqueness and aim of this model. This comment is another reason why I think the title is not match the content.**

Thank you for pointing out that we miss previous literature and do not mention important properties of classical Lotka-Volterra systems. We have added the following text on page 5 line 198, also to clarify that the Lotka-Volterra equations correspond to evolutionary game dynamics:

*“We utilize Lotka-Volterra dynamics to describe our system, developed independently by Alfred Lotka and Vito Volterra in the 1920s. Since then, many extensions of this model have been developed and its equivalence with replicator dynamics with an additional player was proven (Hofbauer, 1981). Mathematical properties of the classical Lotka-Volterra system show either cyclic oscillation or divergent extinction of one species (May, 1972). In any closed Lotka-Volterra system, the predators will eventually die out with the extinction of the prey. This means that the persistent predator-prey systems, without additional stabilizing mechanisms, should exhibit cyclic oscillations. Here we analyze how in cancer, the additional assumption that cancer cells play coordination games impact their eco-evolutionary dynamics and treatment options. As in most models of cancer growth, the focus of our simulations will be on short-run growth rather than long-run dynamics.”*

As in the paragraph, we note here that, in many mathematical models of cancer growth, the system’s long-run dynamics play little role; as soon as the tumor grows near carrying capacity, the patient’s disease is assumed to have progressed. As most growth happens when the tumor is coordinated on either of the two strategies, in our model, progression is deeply tied with coordination. Perhaps this, the nature of the coordination games as we model them in cancer, explains why the reviewer felt we ignore some of the common or basic features of Lotka-Volterra models, many of which study long-run competition.

We also thank you for pointing out that the manuscript needs to do a better job in terms of highlighting the unique aspects of our model. We have added a paragraph in our discussion section to explain these. There are two: introducing the evolutionary game explicitly to the Lotka-Volterra equations’ growth parameter and the convexity parameter. The added paragraph on page 10, line 326 reads:

*“In capturing coordination games in cancer, our paper introduces two technical novelties to Lotka-Volterra competition models. First, we replace the exogenous growth parameter ‘R’ with endogenous growth derived from an evolutionary coordination game. Second, we scale the punishment of discoordination through the convexity parameter (a biological interpretation of which is provided in SI. 3). As a result, most cancer growth occurs when the tumor is coordinated on either of the two strategies (Fig. 1).”*

Comment 5.

**It is difficult to understand the meaning of figures. In Fig.1,**

We thank you for bringing our attention to our error in the manuscript’s figure caption which caused the problems with Figure 1.

Comment 5-1

**the author wrote “The heat map of tumor growth modulated by a pure coordination game”, what is “tumor growth”? Is this “x[t=T]/x[t=0]” when the final time T?**

Tumor growth is the value of $\dot{x}=\dot{x}_{1}+\dot{x}_{2}$ for any given size-composition pair.

Comment 5-2

**I cannot see two red dots in fig.1.**

This is due to the mistake in the figure caption. The ‘red dots’ part was meant for Figure 2.

Comment 5-3.

**Are the vertical and horizontal axes means initial values?**

Figures 1, 2, 3, and 6 don’t feature any dynamics. Figures 2, 3, and 6 are standard phase diagrams. Figure 1 shows the value of tumor growth, $\dot{x}$ for any given size-composition $\left( x,g \right)$ pair.

Comment 5-3.

**What's going on with m1 and m2? (maybe m1=m2=0?)**

Their value is indeed missing from Figure 2. For Figure 1, the values of $m_{1}$ and $m_{2}$ don’t matter as they don’t change tumor growth. We nevertheless added them to Figure 1 as well for completeness.

Comment 5-4.

**The red part on the right is the x1 mountain and the red part on the left is the x2 mountain, is it right? I didn't know this until I did the calculation myself. I'm not sure if it's okay to show two different types of cancer growth in the same system of colors.**

Figure 1 shows total tumor growth. Total tumor size is the only prognosis-relevant metric in our model. In applications it is very rare to observe the growth of different cancer phenotypes within a patient. The figure is there to illustrate that if cancer growth is indeed modulated by a coordination game, growth happens mostly for coordinated tumors.

The caption of Figure 1 on page 6 now reads:

*“The heat map of tumor growth,* $\dot{x}=\dot{x}_{1}+\dot{x}_{2}$*, for the possible size-composition* $(x,g)$ *pairs where the types' growth rates are modulated by a pure coordination game. Type 1's intrinsic growth rate,* $r_{1}$*, is larger and its death rate,* $c_{1}$*, is lower, therefore its fitness peak is higher, but a pure type 2 tumor also has a fitness peak. Parameters:* $r_{1}=0.25, r_{2}=0.17, d_{1}=d_{2}=0, K=100, T=10, \alpha=2,c_{1}=0.05, c_{2}=0.1, m_{1}=m_{2}=0.01.$*”*

References: (references in red have been added to the manuscript.

Andrei, L., Kasas, S., Garrido, I.O., Stankovic, T., Korsnes, M.S., Vaclavikova, R., Assaraf, Y.G., and Pesic, M., 2020. Advanced technological tools to study multidrug resistance in cancer. *Drug Resistance Updates*, 48: 100658.

Arefin, M.R., Kabir, K.A., Jusup, M., Ito, H. and Tanimoto, J., 2020. Social efficiency deficit deciphers social dilemmas. *Scientific Reports*, 10: 1-9.

Eigen, M., Schuster, P. : The hypercycte - a principle of natural self-organization. Berlin, Heidelberg, New York: Springer 1979

Greaves, M., and Maley, C.C., 2012. *Clonal evolution in cancer*. Nature, 481: 306-313.

Han, J., Jun, Y., Kim, S.H., Hoang, H.H., Jung, Y., Kim, S., Kim, J., Austin, R.H., Lee, S., and Park, S., 2016. *Rapid emergence and mechanisms of resistance by U87 glioblastoma cells to doxorubicin in an in vitro tumor microfluidic ecology*. Proceedings of the National Academy of Sciences, 113: 14283-14288.

Hofbauer, J., Schuster, P., Sigmund, K.: A note on evolutionary stable strategies and game dynamics. *J. Theor. Biol*. 81,609-612 (1979)

Hu, Z., Sun, R., and Curtis, C., 2017. A population genetics perspective on the determinants of intra-tumor heterogeneity. *Biochimica et Biophysica Acta (BBA)-Reviews on Cancer*, 1867: 109-126.

Hofbauer, J., 1981. On the occurrence of limit cycles in the Volterra-Lotka equation. *Nonlinear Analysis: Theory, Methods & Applications,* 5(9), pp.1003-1007.

Ito, H. and Tanimoto, J., 2018. Scaling the phase-planes of social dilemma strengths shows game-class changes in the five rules governing the evolution of cooperation*. Royal Society Open Science*, 5: 181085.

Liguoro, D., Fattore, L., Mancini, R., and Ciliberto, G., 2020. Drug tolerance to target therapy in melanoma revealed at single cell level: What next? *Biochimica et Biophysica Acta (BBA)-Reviews on Cancer*, 1874: 188440.

May, R.M., 1972. Limit cycles in predator-prey communities. Science, 177: 900-902.

Maynard-Smith, J., Price, G.R. : The logic of animal conflict. Nature (London) 246, 15-18 (1973)

Maynard-Smith, J. : The theory of games and the evolution of animal conflicts. *J. Theor. Biol*. 47, 209-221 (1974)

Maynard-Smith, J. : Evolution and the theory of games. Cambridge : Cambridge University Press 1982

Schuster, P., Sigmund, K., Wolff, R.: Mass action kinetics of selfreplication in flow reactors. J. Math. Anal. Appl. 78, 88-112 (1980)

Schuster, P., Sigmund, K., Hofbauer, J., Wolff, R. : Selfregulation of behaviour in animal societies*. Biol. Cybern.* 40, 1-8 (1981)

Schuster, P., Sigmund, K. : Replicator dynamics. *J. Theor. Biol*. 100, 533-538 (1983)

Taylor, P., Jonker, L. : Evolutionarily stable strategies and game dynamics*. Math. Biosci*. 40, 145-156 (1978)

Wang, Z., Kokubo, S., Jusup, M., and Tanimoto, J., 2015. Universal scaling for the dilemma strength in evolutionary games. Physics of Life Reviews, 14: 1-30.

Wang, C., Yang, J., Luo, H., Wang, K., Wang, Y., Xiao, Z.X., Tao, X., Jiang, H., and Cai, H., 2020. CancerTracer: a curated database for intrapatient tumor heterogeneity*. Nucleic acids research*, 48: D797-D806.

Wölfl, B., te Rietmole, H., Salvioli, M., Kaznatcheev, A., Thuijsman, F., Brown, J.S., Burgering, B. and Staňková, K., 2021. The contribution of evolutionary game theory to understanding and treating cancer. *Dynamic Games and Applications*, 1-30.
